# Comparative study of grab, DGT, and bryophyte sampling as monitoring program for quality management: case of arsenic in freshwater

**DOI:** 10.1007/s11356-025-36470-9

**Published:** 2025-05-07

**Authors:** Juliette Rougerie, Stéphane Simon, Patrice Fondaneche, Iris Perez-Salva, Laurent Palfner, Jean-Pierre Rebillard, Luc Barbe, Christine Fabry, Gilles Guibaud

**Affiliations:** 1 University of Limoges, E2Lim, 123 Avenue Albert Thomas, Limoges Cedex, 87060 France; 2 Eau Grand Sud-Ouest, CS 87 801, 90 R. du Férétra, Toulouse Cedex 4, 31078 France; 3 OFB - Direction Régionale Occitanie, 90 R. du Férétra, Toulouse, 31400 France

**Keywords:** Arsenic, Water monitoring, Bryophyte indicators, DGT passive sampling, Fractionation, Redox speciation

## Abstract

Selecting the most appropriate method for monitoring arsenic in freshwaters is crucial due to the diversity of available approaches and their inherent objectives, advantages, and limitations. This study addresses this challenge by conducting a comparative analysis of three well-established monitoring methodologies that have been among the most used in the last 30 years. Grab sampling, passive sampling using diffusive gradients in thin films (DGT), and bryophyte sampling were selected to monitor arsenic in a main river and its tributaries over a full hydrological year. On the one hand, grab and passive samplings both indicate stable concentrations of dissolved arsenic throughout the monitoring period and across the whole studied area. They also highlight a stable fractionation and redox speciation of arsenic, predominantly present as labile As(V). On the other hand, bryophyte monitoring exhibits significant spatiotemporal variations of arsenic content, with differences reaching up to tenfold. These contents are not solely determined by arsenic occurrence in water but result from arsenic bioavailability and its bioaccumulation in the organisms, which are both influenced by environmental factors and uptake mechanisms. Thus, the results of this study highlight the importance of clearly stating the goals of a monitoring programme in order to identify the most suitable method and implementation, ensuring relevant environmental management decisions.

**Supplementary Information:**

The online version contains supplementary material available at 10.1007/s11356-025-36470-9.

## Introduction

Arsenic is a naturally occurring element that can be found in surface waters due to weathering of minerals or to human activities such as mining and agriculture (Mandal and Suzuki 2002). Arsenic is notorious for its complex speciation which may pose significant challenges to monitor its presence in natural waters. Indeed, it exists predominantly as inorganic oxyanions, including trivalent arsenite (As(III)) and pentavalent arsenate (As(V)) (Smedley and Kinniburgh 2002; Sharma and Sohn 2009). The speciation of arsenic is influenced by redox conditions, with As(V) being thermodynamically stable in oxic environments while As(III) predominates in reduced conditions (Cullen and Reimer 1989). In addition, As(III) is generally considered more toxic and readily absorbed by organisms due to its higher solubility and ability to passively diffuse through biological membranes (Smedley and Kinniburgh 2002). For an accurate toxicity risk assessment, a quantitative determination of its most reactive species is therefore essential. However, in conventional regulatory surveys, its redox speciation is often overlooked, and its behavior is sometimes mistakenly assumed to be similar to cationic metals during water quality data processing.

The assessment of arsenic contamination in freshwater necessitates the establishment of measurement networks. They are required by the Water Framework Directive (WFD 2000/60/CE) to evaluate the contamination status of watercourses across Europe, but also at the national or local level in rehabilitation programs to monitor the water quality and its improvement. Among the available approaches for arsenic monitoring, three methods are commonly employed: grab sampling, passive sampling by diffusive gradients in thin films (DGT), and the use of biological indicators such as bryophytes.

Grab sampling involves collecting water samples at discrete time points. This method offers simplicity and ease of implementation, making it widely used in routine regulatory monitoring programs. However, relying on low-frequency sampling may overlook temporal variations and fail to capture short-term changes in arsenic levels. Additionally, grab sampling may introduce issues related to sample preservation during storage and transportation, particularly resulting in speciation evolution, potentially leading to inaccuracies in assessment and data interpretation.

The DGT is a non-biological integrative passive sampling technique, developed in 1994 (Davison and Zhang 1994), that allows the determination of a DGT-labile fraction, i.e., the quantification of the element in its free form or associated in complexes with rapid dissociation kinetics. This fraction is considered a biologically more relevant fraction than the dissolved fraction (Menegário et al. 2017). Some specific DGT configurations can also be used to perform an in situ discrimination of the chemical species, giving access to an assessment of the speciation. In addition, the DGT determines a time-weighted average concentration (usually over a few weeks) ensuring a better temporal representativeness of the contamination than grab sampling, especially in dynamic systems with evolving concentrations and speciation (Røyset et al. 2005; Eismann et al. 2020). Nevertheless, DGT deployment requires careful consideration of factors such as deployment conditions (e.g., water temperature, water flow, and biofilm presence) (Gimpel et al. 2001; Uher et al. 2012; Devillers et al. 2017a) and calibration (e.g., use of an appropriate diffusion coefficient) (Bennett et al. 2010; Ding et al. 2016).

Bryophytes have been used for several decades as indicators of pollutant presence in surface waters (Gecheva and Yurukova 2014). Due to their ability to absorb and accumulate pollutants from their environment, resulting in concentrations several times higher than in the surrounding environment (Wehr and Whitton 1983; Zechmeister et al. 2003), they allow detecting the presence of pollutants at lower levels than traditional grab sampling. In addition, due to their life span of up to several years (During 1979), they can be used to monitor changes in concentrations over time, at the scale of several months or years. However, several studies have failed to find a clear link between the contents in bryophytes and the concentrations in water. For example, Vazquez et al. (2004) found that, for 17 studied elements over 22, the concentrations in water were poorly correlated to the contents in *F. antipyretica* collected at the same points. This may result from a limited evaluation of the concentration in water. Indeed, in most cases, only one or two sampling points are monitored by grab sampling (Debén et al. 2015) at the moment of bryophyte sampling, leading to a lack of temporal representativeness of the grab sample. Furthermore, generally only the total dissolved metal concentration is determined, which may not correspond to the bioavailable fraction of metals (Tessier and Turner 1995). Indeed, the uptake rate by bryophytes may vary depending on both fractionation and speciation of the metals/metalloids. As an example, the accumulation of copper in *F. antipyretica* is more strongly correlated to weakly complexed copper concentrations, rather than to free copper concentrations (Bourgeault et al. 2013). Aquatic bryophytes have already been used to monitor arsenic in water quality assessment (Debén et al. 2015). The ability of the genus *Fontinalis* to absorb arsenic in rivers with urban, industrial, and agricultural context has been particularly highlighted (Tipping et al. 2008; Favas et al. 2012).

The aim of this study was to compare these three well-established approaches for the monitoring of arsenic in a watercourse system composed of a main river and three of its tributaries, over a complete hydrological year. The evaluation of the information obtained with each tool, its relevance, and their potential complementarity will provide insights on how to select the most suitable arsenic monitoring method according to specific monitoring goals or environmental contexts.

## Materials and methods

### Study area

**Fig. 1:**
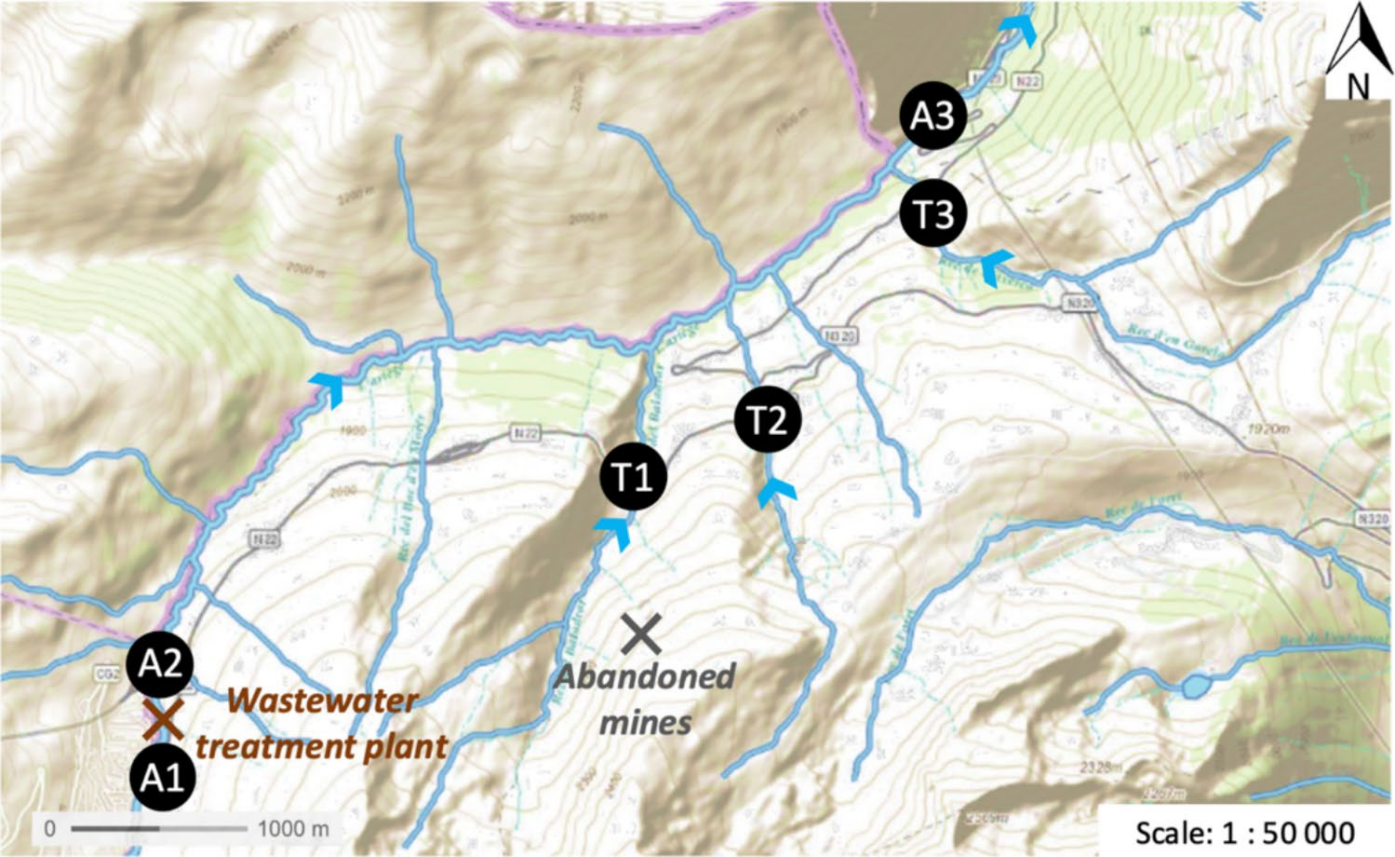
Temporal evolution of total, dissolved and labile arsenic concentrations over the one-year monitoring at point T2 and A3 (mean value and standard deviation, *n* = 2)

The studied area is a mountainous river with a torrential flow located, located in the south-west of France, at an altitude between 1500 and 2100 m. Six sampling points were selected: three situated along the main river under investigation (A1, A2, and A3) and three along its tributaries (T1, T2, and T3). Their exact location was chosen in order to ensure a significant flow all throughout the year to limit the impact of the double boundary layer on DGT measurement as well as sediments or biomass accumulation in the vicinity of the devices. Potential sources of arsenic within this area comprise the geochemical background, characterized by plutonic rock formations, as well as abandoned iron mines and a discharge from a wastewater treatment plant (Fig. 1). The freshwater physicochemical characteristics of each site are detailed in Table S1.

### Monitoring of arsenic

Arsenic was monitored using grab, DGT, and bryophyte sampling over a full hydrological year, between October 2020 and October 2021. For each of the six sampling points, DGT samplers were deployed throughout the year for 14-day periods, and grab samplings were performed simultaneously to every DGT deployments. In addition, bryophyte samplings were performed at two different seasons (February 2021 and September 2021), as recommended by Deben et al. (2015). These frequencies correspond to what is usually performed in regulatory programs, except for grab sampling which was performed more frequently (up to 26 samplings per site instead of usually only 1 to 12 per year).

#### Grab sampling

Grab samples were taken by immersion of polyethylene bottles to determine the total arsenic. Fifteen milliliters was immediately filtered in situ with a cellulose acetate syringe filter (pore diameter 0.45 μm) and then acidified with 2% (V/V) HNO_3_ to determine the dissolved fraction of arsenic. Another 10 mL aliquot was filtered and acidified with 1% (V/V) HCl to assess arsenic speciation. To highlight any speciation changes during transport and storage, mostly the conversion of As(III) to As(V), some grab samples were spiked with known amounts of As(III) during sampling. Each sampling was performed in duplicate. Reagents with analytical grade or higher were used to avoid any arsenic contamination during the sample preparation. The absence of contamination was verified by performing blanks with ultrapure water submitted to the same pre-treatment on the field than freshwater samples. All the samples were brought back to the laboratory in an ice box and then stored at 4 °C until analysis. All analysis (see “Analytical methods” section) were performed within one week after sampling to avoid any evolution in arsenic speciation.

#### DGT sampling

A conventional DGT device is composed of three layers, i.e., a binding layer that irreversibly traps the targeted analytes in environmental conditions, a diffusive gel layer that allows a controlled diffusion of the analytes within the device and a filter membrane that protects the diffusive layer from damage by particles in the environment. All these layers are housed in a plastic holder with a 3.14 cm^2^ sampling area (Zhang and Davison 1995). In this study, two setups of DGT were used for arsenic monitoring: one with a 3-mercaptopropyl-functionalised silica binding phase for a selective As(III) determination and one with a zirconium oxide binding gel for total labile arsenic, labelled DGT-Th and DGT-Zr respectively. The DGT-Th were purchased from DGT Research Ltd. The zirconium oxide binding gels were prepared at the laboratory according to the protocol developed by Guan et al. (2015) with modifications by Devillers et al. (2017a, b). The DGT-Zr devices were assembled using plastic holders (DGT Research Ltd.) enclosing a binding gel, a polyacrylamide diffusive gel (0.077 cm thickness, prepared according to the protocol used by Zhang et al. (1998)), and a polycarbonate filter membrane (0.4 μm pore diameter, 0.01 mm thick, Whatman).

At each campaign, two DGT-Th and two DGT-Zr were simultaneously deployed for 14 days, except for point A1 that was not monitored by DGT. Temperature loggers (Tynitag) were also deployed to record the water temperature every 10 min during the DGT exposure. After 14 days, the DGTs were brought back to the laboratory in an ice box and stored at 4 °C. Within 48 h, the DGTs were dismantled, the binding gels were rinsed with ultrapure water and eluted at 20 ± 1 °C in a temperature-controlled room. The 3-mercaptopropyl-functionalised silica and zirconium oxide binding gels were eluted for 24 h with 2 mL of 1 mol L^−1^ HNO_3_/0.01 mol L^−1^ KIO_3_ and 2 mL of 0.005 mol L^−1^ NaOH/0.5 mol L^−1^ H_2_O_2_ respectively. The eluates were stored at 4 °C until analysis by inductively coupled plasma-mass spectroscopy (ICP-MS) Agilent 7700X to determine the accumulated mass of arsenic (see “Analytical methods” section). This accumulated mass was used to calculate the time-weighted average concentration in water over the 14-day sampling period (*C*_W_) according to the Eq. 1 (Davison and Zhang 1994):

1 $$C_w=\frac{m\triangle g}{D\;A\;t}$$

where *m* (ng) is the accumulated mass of arsenic, ∆*g* is the thickness of the diffusive layer and polycarbonate filter (0.078 cm), *t* (s) is the deployment duration, *A* is the exposure area (3.14 cm^2^), and *D* (cm^2^ s^−1^) is the diffusion coefficient of arsenic in the diffusive gel. The values of D taken from literature were corrected according to the average temperature during the deployment using the Stokes–Einstein relationship. The *D* value for As(III) (9.0 × 10^−6^ cm^2^ s^−1^ at 25 °C) was taken from Bennett et al. (2010), and the *D* value for As(V) (6.9 × 10^−6^ cm^2^ s^−1^ at 25 °C) from Ding et al. (2016) was used for the total labile As sampled by the DGT-Zr as the major part of As (> 70%) was As(V).

The absence of arsenic contamination of the DGT monitoring was verified by performing DGT-Th and DGT-Zr blanks. At each campaign, two DGT of each type were taken on a sampling point and exposed to the air but not deployed in the river. They were then brought back to the laboratory and stored at 4 °C for the rest of the field exposure period. They were dismantled with the exposed DGT devices in a random position.

#### Bryophyte sampling

In the absence of standardized procedure, the recommendations proposed by Debén et al. (2015) were followed for bryophyte monitoring which correspond to the usual procedure applied by regulatory freshwater monitoring programs in France. Only fully submerged plants were sampled over a segment of about 10 m. The plant material was washed in the river to remove particles and kept in clean plastic bags at 4 °C. The bryophytes were identified and prepared at the laboratory within 3 days. After drying in an oven at 40 °C for 24 h to limit the loss of arsenic by volatilization, green shoot subsamples taken at each sampling sites were pooled then ground in an agate mortar and submitted to microwave-assisted acid digestion. A total of 250 mg of sample was placed in a Teflon digestion tube with 2 mL of H_2_O_2_ (30%), 6 mL of HNO_3_ (65%), and 3 mL of HCl (37%). The mixture was placed in a microwave oven (Multiwave Go, Anton Paar) at 180 °C for 40 min. The volume was then made up to 50 mL with ultrapure water in a calibrated flask. All samples were prepared in duplicate, and digestion blanks were also performed without plant material. The bryophyte arsenic contents were expressed as mg of arsenic by kg of dried mass of biological material.

### Analytical methods

Arsenic concentrations in grab samples, DGT eluates, and bryophyte samples were determined by ICP-MS (Agilent 7700X). All the analyzed solutions were acidified to 2% (V/V) HNO_3_. Indium (^115^In) was used as internal standard. The stability of the ICP-MS measurements was checked with a control solution (10 µg L^−1^) every ten samples. The accuracy was verified using a river water reference material (SLRC-5 from the National Research Council, Canada). The limit of quantification was 0.02 µg L^−1^.

Arsenic speciation in filtered grab samples was assessed by liquid chromatography coupled to atomic fluorescence spectrometry (LC-AFS) with hydride generation (HG) according to Wan et al. (2014). The chromatographic separation was performed using a Hamilton PRP-X100 column with a phosphate solution as mobile phase (25 mmol L^−1^ NH_4_H_2_PO_4_ and 3 mmol L^−1^ Na_2_SO_4_) at a flow rate of 1 mL min^−1^. The HG was conducted using solutions of 0.37 mol L^−1^ NaBH_4_ with 0.1 mol L^−1^ NaOH and 3 mol L^−1^ HCl, which were injected at a flow rate of 0.8 mL min^−1^. Arsenic was detected by atomic fluorescence spectrometry (PSA Analytical EXCALIBUR®). The limits of quantification were 0.2 and 0.4 μg L^−1^ for As(III) and As(V) respectively.

### Statistical analysis

The results are presented in the form of mean ± standard deviation. Comparison tests (either Student’s *t*-test or ANOVA) were conducted at the significance level of *α* = 0.05.

## Results and discussion

All the monitoring results obtained over the 1-year monitoring at each sampling point are presented in Figure S1. On the one hand, the combination of grab and DGT samplings is expected to determine the total, dissolved, and labile arsenic fractions and assess dissolved arsenic speciation. On the other hand, bryophyte monitoring is expected to determine the bioavailable arsenic fraction.

### Arsenic fractionation

**Fig. 2:**
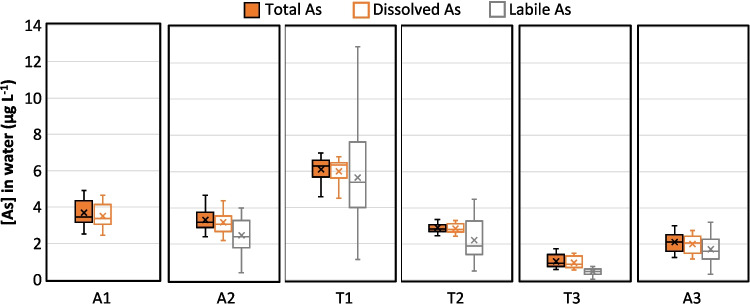
Dispersion of the percentage of As(V) in the dissolved and DGT labile arsenic fraction over the one-year monitoring at each sampling point. The concentrations of As(III) and (V) were below the detection limit in grab samples for point T3 and were not monitored by DGT on point A1 (evaluation based on n= 44 to 50 values)

**Fig. 3:**
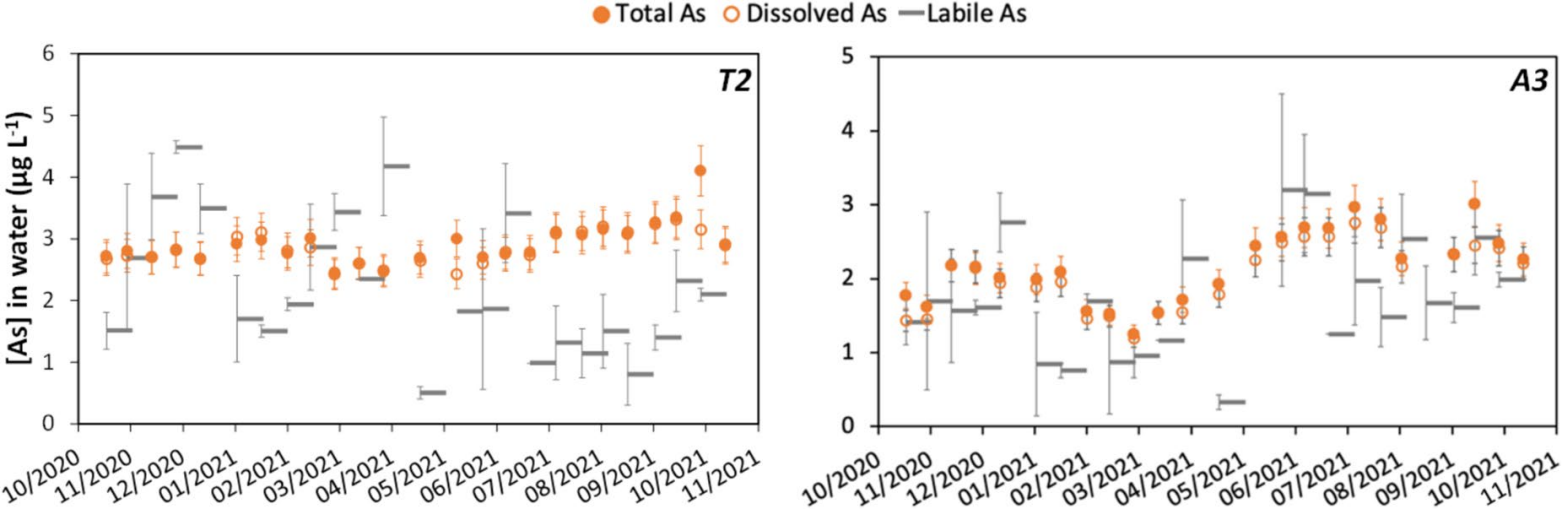
Temporal evolution of As(III) concentration monitored by grab and DGT sampling at point T2 (mean value and standard deviation, *n* = 2)

The total arsenic concentrations obtained by grab sampling are of the same order of magnitude throughout the study area with average concentrations generally between 1 and 4 µg L^−1^ (Fig. 2). The concentration in the first tributary (sampling point T1) appears to be slightly higher, with an average concentration of 6 µg L^−1^ that could potentially be explained by the presence of abandoned mines in its catchment area (Fig. 1). Conversely, the third tributary (sampling point T3) displays lower arsenic total concentrations, between 0.6 and 1.7 µg L^−1^. For all sampling points, the 1-year monitoring revealed that the total arsenic concentrations are overall stable over time, with less than 35% variation around the mean value, as illustrated in Fig. 3 for point T2. Only a slight seasonal effect seems to be highlighted at points T1, T3, and A3 with a 30% drop in concentrations in March. Since the sites T1 and T3 are contributors to site A3, the observation of a seasonal effect on these three sites would seem consistent.

The relative constant total concentration does not necessarily imply that arsenic bioavailability is constant, since it strongly depends on the fractionation, particularly the dissolved and labile fractions, and the speciation of arsenic.

The filtration performed during the grab sampling allowed the measurement of the dissolved arsenic fraction that includes both labile and non-labile dissolved fractions. No significant differences were observed between the total and dissolved concentrations for each sampling point, with dissolved arsenic representing more than 92% of total arsenic. The very few exceptions to this trend (6 on 147 samplings) could potentially be attributed to sample contaminations. The high degree of concordance between dissolved and total concentrations, with respect to both their values and temporal evolution, suggests that the arsenic present in the studied watercourses is entirely in soluble form.

The DGT results exhibit higher uncertainties than grab sampling, with relative standard deviations (RSD) between duplicates ranging on average from 15 to 30%. Points T3 and A3 displayed notably high uncertainties, with RSD reaching 140% between duplicates (Figure S1). During this study, elevated uncertainties were due to unfavorable deployment conditions, with very turbulent flows (Figure S2) that induced the intrusion of particles inside some devices (DGT sealing issue). Putting aside these exceptions, DGT enables a reliable estimation of labile arsenic concentrations with a precision of 1 µg L^−1^. The estimated labile arsenic concentrations exhibit a high degree of similarity with the dissolved concentrations. Indeed, for all the sampling points, the labile concentrations are either not significantly different or slightly lower than the dissolved concentrations in most samples. Only a few measurements (1 to 3 cases for points A2, T1, and T2) were inconsistent, with labile arsenic concentrations 60 to 100% higher than the dissolved concentrations (Figure S2). These discrepancies may be attributed to DGT sealing issues or to an increase of arsenic concentration during the 14-day deployment that was integrated by the DGT but not monitored by the low-frequency grab sampling. Overall, the evolution of dissolved and labile concentration over time indicates the same trend as illustrated on Fig. 3 for points T2 and A3. The similarities between the DGT measurements and the dissolved fraction thus indicate that dissolved arsenic is predominantly labile.

### Arsenic speciation

**Fig. 4:**
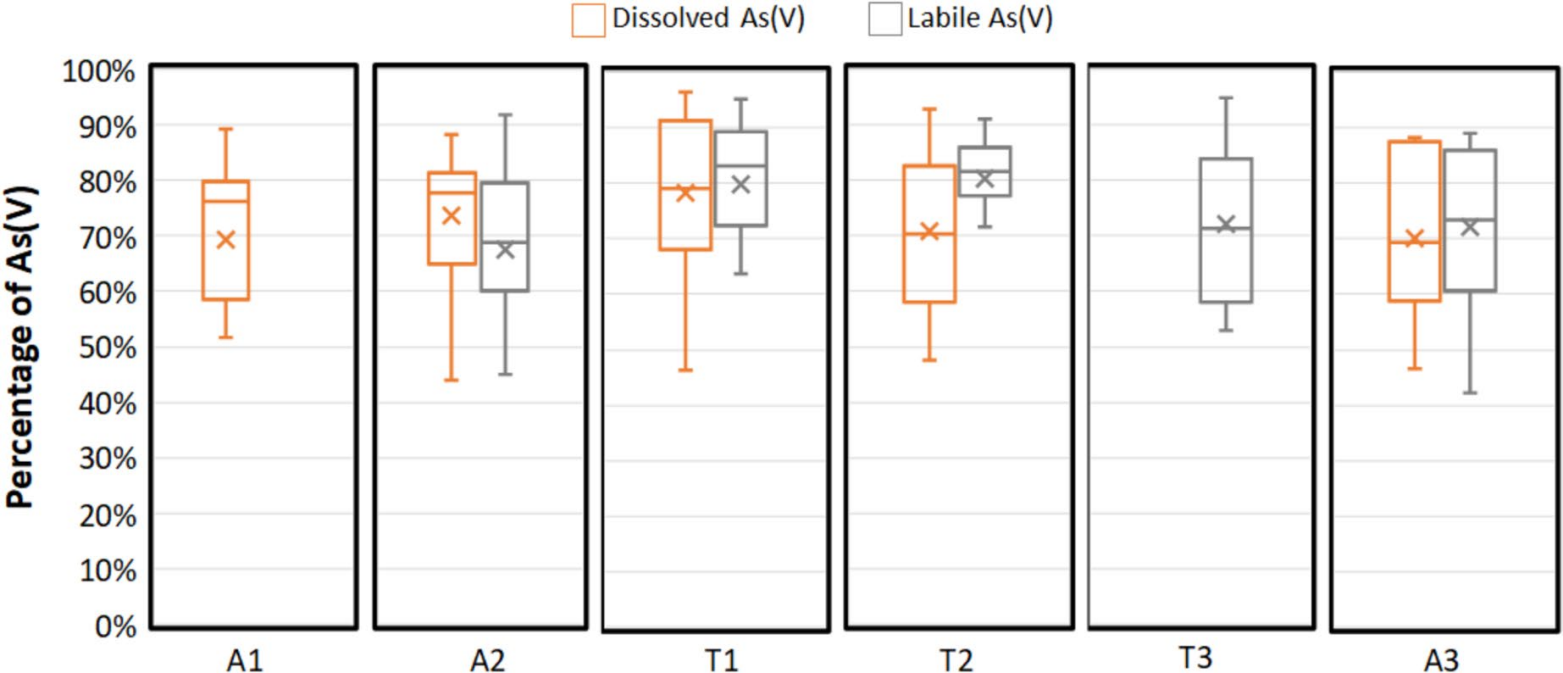
Dispersion of the percentage of As(V) in the dissolved and DGT labile arsenic fraction over the one-year monitoring at each sampling point. The concentrations of As(III) and (V) were below the detectionlimit in grab samples for point T3 and were not monitored by DGT on point A1 (evaluation based on *n* = 44 to 50 values)

**Fig. 5:**
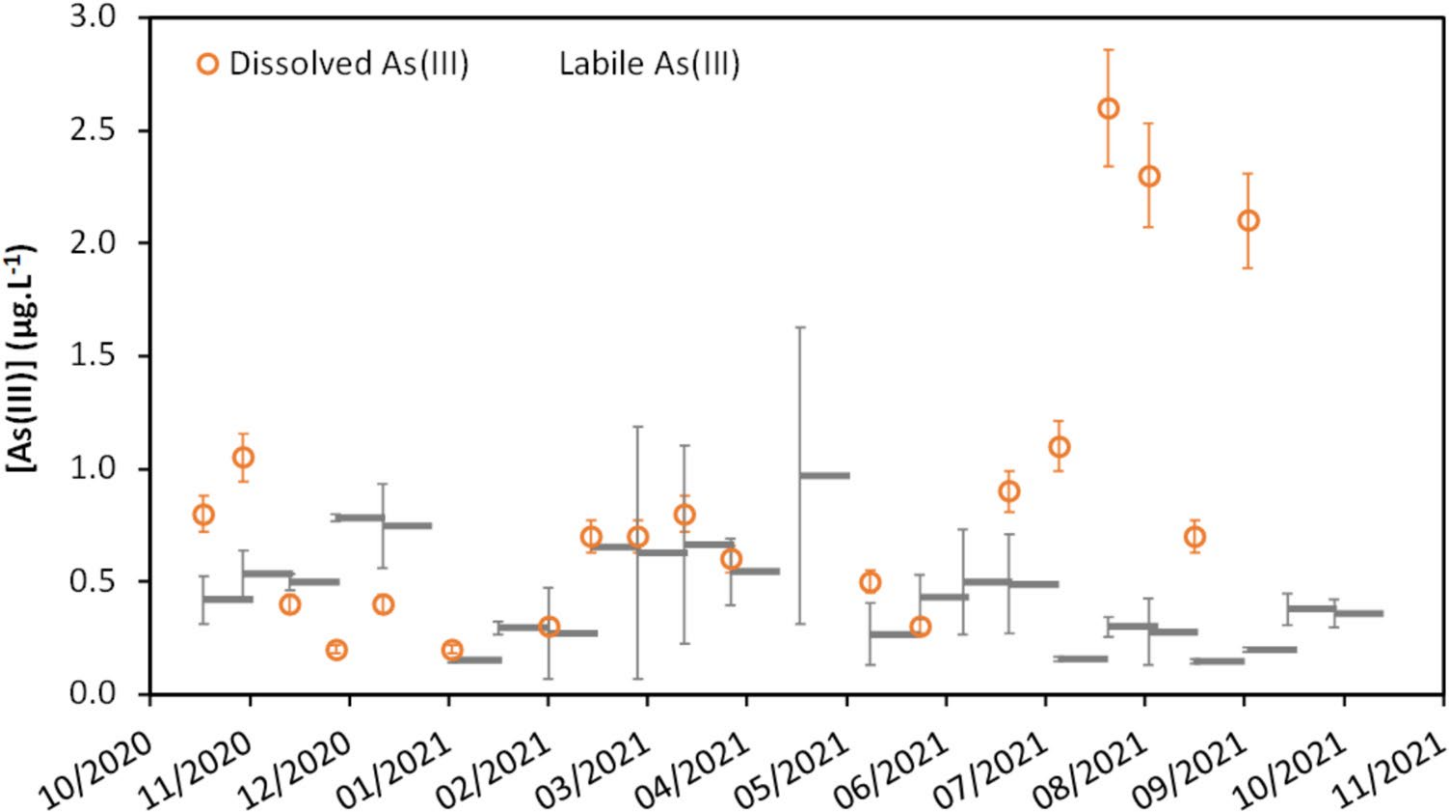
Temporal evolution of As(III) concentration monitored by grab and DGT sampling at point T2 (mean value and standard deviation, *n* = 2)

The arsenic speciation, i.e., distribution between As(III) and As(V), was determined by both grab and DGT sampling. Analysis of grab samples spiked with a known amount of As(III) during sampling did not show any conversion to As(V). In contrast to grab sampling, DGT-Th accumulates As(III) in situ, thus minimizing the risk of interconversion. Over the 1-year monitoring, both grab and DGT sampling reveal that, at each sampling point, As(V) represents, in average, more than 70% of arsenic (Fig. 4). These results are consistent with O_2_ saturation in surface freshwaters, corresponding to oxidizing conditions. Regarding the temporal trend (Figure S1), the concentrations of As(III) showed relatively stable patterns over time. An exception was observed for all grab samples between July and August. This phenomenon is illustrated for point T2 in Fig. 5. However, this change of trend was not detected by the DGT measurements, indicating that it could result from speciation evolution during transport and storage. Indeed, some discrepancies were detected between several duplicates of grab sampling during this period, emphasizing the hypothesis of bias in samples preservation.

### Arsenic in bryophytes

**Fig. 6:**
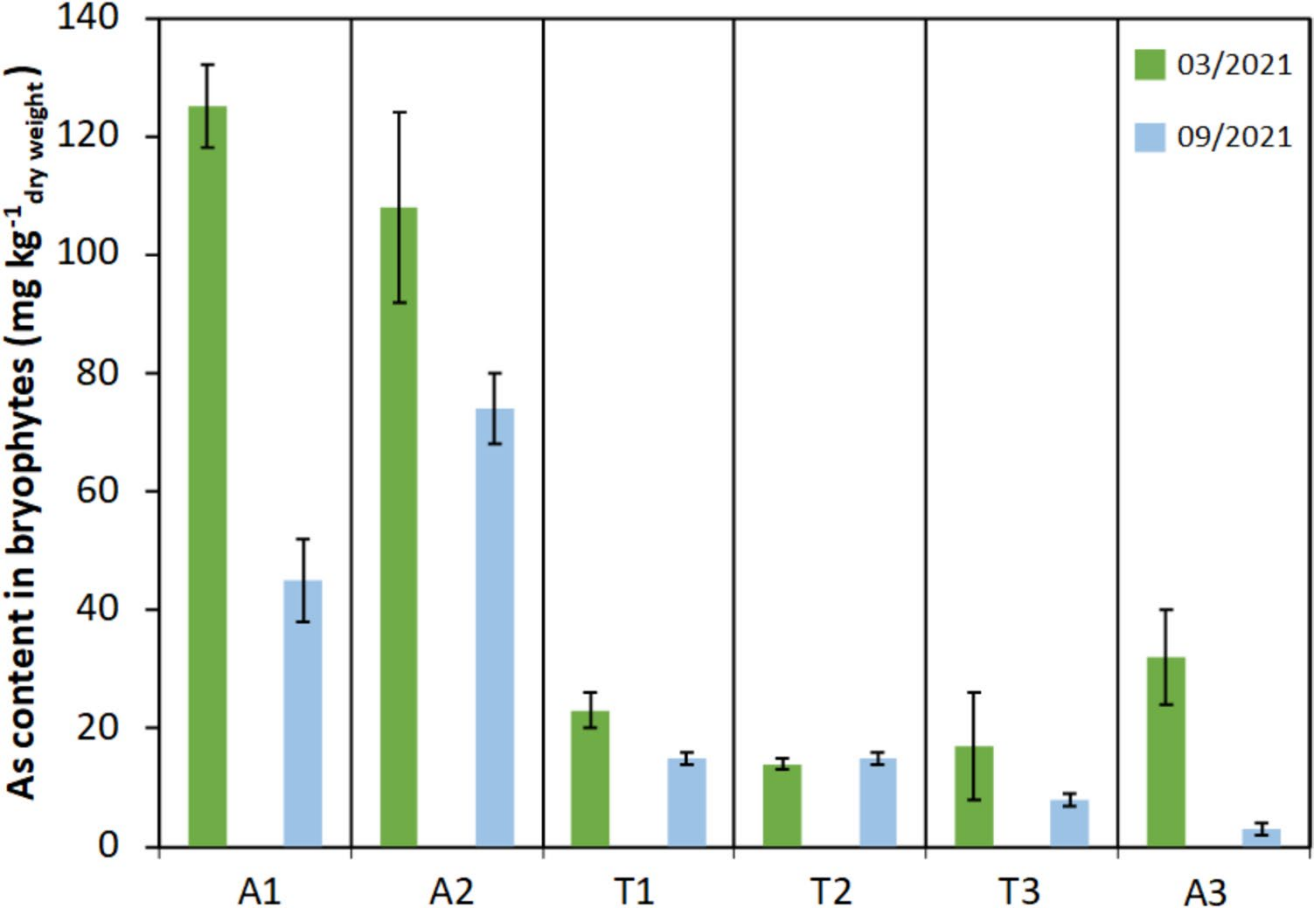
Arsenic content in bryophytes over the two sampling campaigns at each sampling point (mean value and standard deviation, *n* = 2)

The arsenic contents in bryophytes, obtained for each campaign at the six sampling points, are given in Fig. 6. A large range of values was observed, covering nearly two orders of magnitude (between 3 ± 1 and 108 ± 16 mg kg^−1^). For points A1 and A3, a significant (*α* = 0.05) temporal evolution of arsenic contents was observed, with a 3- to tenfold lower content for the second sampling. To a lesser extent, the same trend was also observed for point T1 (*α* = 0.05). Furthermore, significant (*α* = 0.05) differences were observed between the sampling points. Arsenic contents in bryophytes were about 4- to fivefold higher for points A1 and A2 than for the other ones. Thus, based on the assessment of arsenic contamination using bryophytes, the sampling points can be sorted under three groups: one group with significant temporal variations (A1, A3, T1), one with high arsenic content (A1, A2), and one with low content (T1, T2, T3 and A3).

### Comparison of arsenic monitoring by grab, DGT, and bryophyte sampling

In this study, grab sampling, the most used method in regulatory monitoring programs, allowed the evaluation of temporal dynamics of total and dissolved concentration and of arsenic speciation. It highlighted that arsenic was mainly found in the dissolved fraction as As(V). The DGT sampling, which monitors the evolution of labile arsenic and speciation of arsenic based on a 15-day time integration, lead to a similar conclusion, with the additional information that arsenic was mainly labile. Both approaches indicated that arsenic presence remained consistent throughout the year and similar across the entire studied area. In this situation of a stable arsenic occurrence over time, a routine grab sampling with low frequency (2 to 12 times a year) would have likely led to the same conclusions. However, even though a measurement network based on DGT sampling is 2 to 3 times more expensive (Rougerie et al. 2021), it offers the advantage of a time-averaged concentration over 2 weeks, which can be beneficial in case of unexpected variations in concentration. Furthermore, DGT sampling reduces the issue of speciation changes during sample transport and storage, as observed for some grab samplings during the summer period (Fig. 5). The monitoring based on bryophytes, a biological indicator which can give an estimation of the bioavailability, showed significant spatiotemporal variations of arsenic concentrations, which appears inconsistent with the relatively stable patterns obtained with water monitoring (grab and DGT samplings). This discrepancy has been previously highlighted in the literature and was often attributed to a lack of information due to limited water analysis (López and Carballeira 1993; Vazquez et al. 2004; Favas et al. 2018). Indeed, most of the previous studies relied on low-frequency water grab sampling or assessment of total or dissolved arsenic only, without taking into consideration its fractionation nor speciation (Debén et al. 2015). To address this issue, our study specifically employed a more robust approach with frequent water grab samplings, DGT measurements and speciation analysis, which revealed only limited variations in arsenic concentrations, fractionation (total, dissolved, labile), and speciation (As(III) and (As(V)). This suggests that the changes in the arsenic accumulation in bryophytes may be strongly affected by other physicochemical parameters (e.g., pH, organic matter) and/or by characteristics of the organisms.

Due to the chemical similarity between arsenate and phosphate, a vital element for living organisms, arsenate is presumed to be taken up by the phosphate transporters of the bryophytes (Zhao et al. 2010; Niazi et al. 2017). Thus, variations in phosphate concentrations could lead to variations in arsenic accumulation. Phosphate concentrations were monitored during this study but were below the limit of quantification (Table S1), so no information is available to support this hypothesis.

Our study identified four different bryophyte species within the sampling areas (Table 1), each sampling point dominated by a single species, except for point T1 where a mixture of two bryophytes was observed. Previous research has demonstrated that various bryophyte species possess varying capacities for arsenic accumulation (Díaz et al. 2013; Favas et al. 2012). In this study, significantly higher arsenic contents were observed in *Rhynchostegium riparioides* (points A1 and A2), whereas arsenic concentration in water was not significantly different compared to the other points. This may suggest that this species may accumulate more arsenic than others. However, limited information exists regarding the specific accumulation capacity of *Rhynchostegium riparioides* compared to other water moss species. Further research would thus be needed to support this hypothesis. Finally, the accumulated arsenic content could be influenced by the age and growth stage of the collected bryophytes, as suggested by Debén et al. (2015).

**Table 1:** Species of native aquatic bryophytes sampled at each point

Sampling point	Native aquatic bryophyte species
A1	*Rhynchostegium riparioides*
A2	*Rhynchostegium riparioides*
T1	*Cratoneuron commutatum; Jungermannia exsertifolia*
T2	*Cratoneuron commutatum*
T3	*Brachythecium rivulare*
A3	*Brachythecium rivulare*

## Conclusion

A relevant management of freshwater resources requires an effective freshwater monitoring network deployment, particularly when toxic compounds such as metals and metalloids are involved. This study, which takes arsenic as an example of a toxic metalloid with complex chemical behavior in freshwater environments, highlights the importance of understanding the objectives, strengths, and limitations of various well-established monitoring methodologies.

While grab and DGT samplings indicated stable and similar spatiotemporal arsenic patterns (including concentrations, fractionation, and speciation) throughout the river system over 1 year, the bryophyte monitoring revealed considerable variations. This emphasizes the complexity of the relationship between arsenic occurrence and its bioavailability, and thus, the need for multi-faceted monitoring approaches if water quality and biota exposure are to be both evaluated.

To achieve effective management of arsenic contamination in freshwater ecosystems, the following recommendations are proposed:

- The evaluation of arsenic occurrence, in terms of total concentration, fractionation, and speciation, can be efficiently assessed by grab or DGT sampling. Although more expensive, DGT gives average values over 1 or 2 weeks, significantly improving the accuracy compared to a temporal snapshot. This is particularly interesting in the case of very low frequency sampling, like for example as requested in regulatory water quality evaluation.
- A comprehensive understanding of arsenic dynamics would require high-frequency grab sampling. Integrated measurements using consecutive DGT deployments could reduce the sampling effort without compromising data quality.
- The assessment of biota exposition risks requires a monitoring based on bioindicators, such as bryophytes or gammarids (AFNOR Standard XP T90-722–3), which also integrates the environmental factors (e.g., pH, presence of competitors or pollutants) that influence arsenic bioavailability.

## Supplementary Information

Below is the link to the electronic supplementary material.

ESM 1 (DOCX 13.5 MB)

## Data Availability

Not applicable.
