# Supplementary material for: Comparative study of grab, DGT, and bryophyte sampling as monitoring program for quality management: case of arsenic in freshwater
Source: Environ Sci Pollut Res Int. 2025 May 7;32(21):12799–808. doi: 10.1007/s11356-025-36470-9 (PMC12119767; doi:10.1007/s11356-025-36470-9)
Supplement: Supplementary file 1 — (DOCX 13.5 MB) [file 11356_2025_36470_MOESM1_ESM.docx]

**Comparative study of grab, DGT and bryophytes sampling as monitoring program for quality management: case of arsenic in freshwater**

**Authors:** Juliette ROUGERIE^1^, Stéphane SIMON^1^, Patrice FONDANECHE^1^, Iris PEREZ-SALVA^2^, Laurent PALFNER^2^, Jean-Pierre REBILLARD^2^, Luc BARBE^3^, Christine FABRY^3^ and Gilles GUIBAUD^1^

**Affiliations:**

^1^ University of Limoges, E2Lim, 123 Avenue Albert Thomas, 87060 Limoges Cedex, France

^2^ Eau Grand Sud-Ouest, CS 87 801, 90 R. du Férétra, 31078 Toulouse Cedex 4, France

^3^ OFB - Direction régionale Occitanie, 90 R. du Férétra, 31400 Toulouse, France

***Corresponding author:** stephane.simon@unilim.fr

**Supplementary material**

**Contents**

[*Table S1: Freshwaters physico-chemical parameters in the study area over the one-year monitoring. 2*](file:////Users/julietterougerie/Desktop/Postdoc/DGT:Bryophytes%20As/Rédaction%20Article/Sup%20Mat_%20Publi%20Ariege%20As_Vf.docx#_Toc140739410)

*Figure S1: Evolution of arsenic fractionation and speciation (grab and DGT sampling) over the one-year monitoring and arsenic contents in bryophytes at each sampling site…………………… 3*

*Figure S2: Unfavorable deployment conditions: low water level, strong current, and DGT placed on a sandy bed, leading to the intrusion of particles between the diffusive and binding gel……..5*

**Table S1: Freshwaters physico-chemical parameters in the study area over the one-year monitoring (n = 44 to 50 for As concentrations and n=22 to 25 for other measurements).**


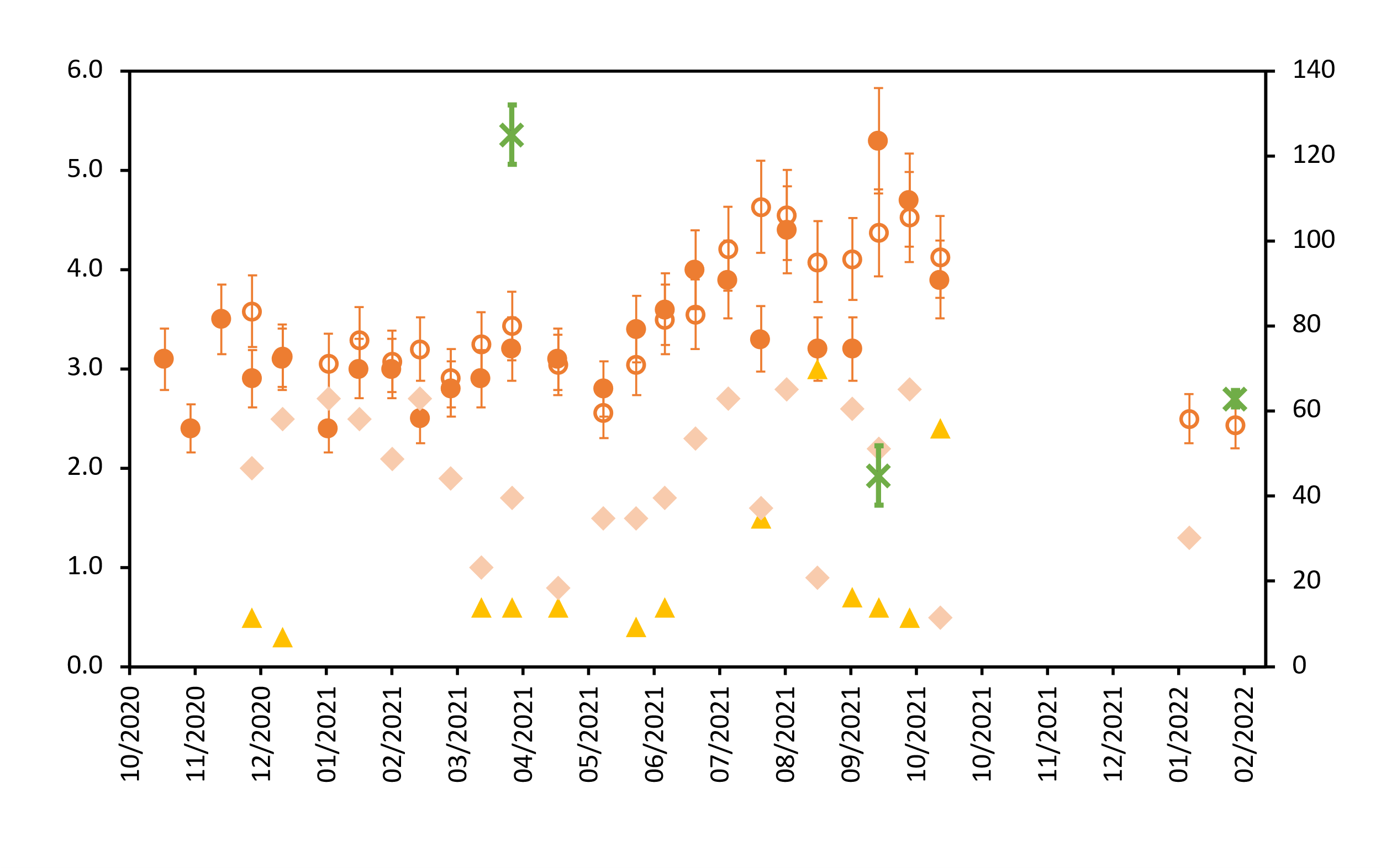

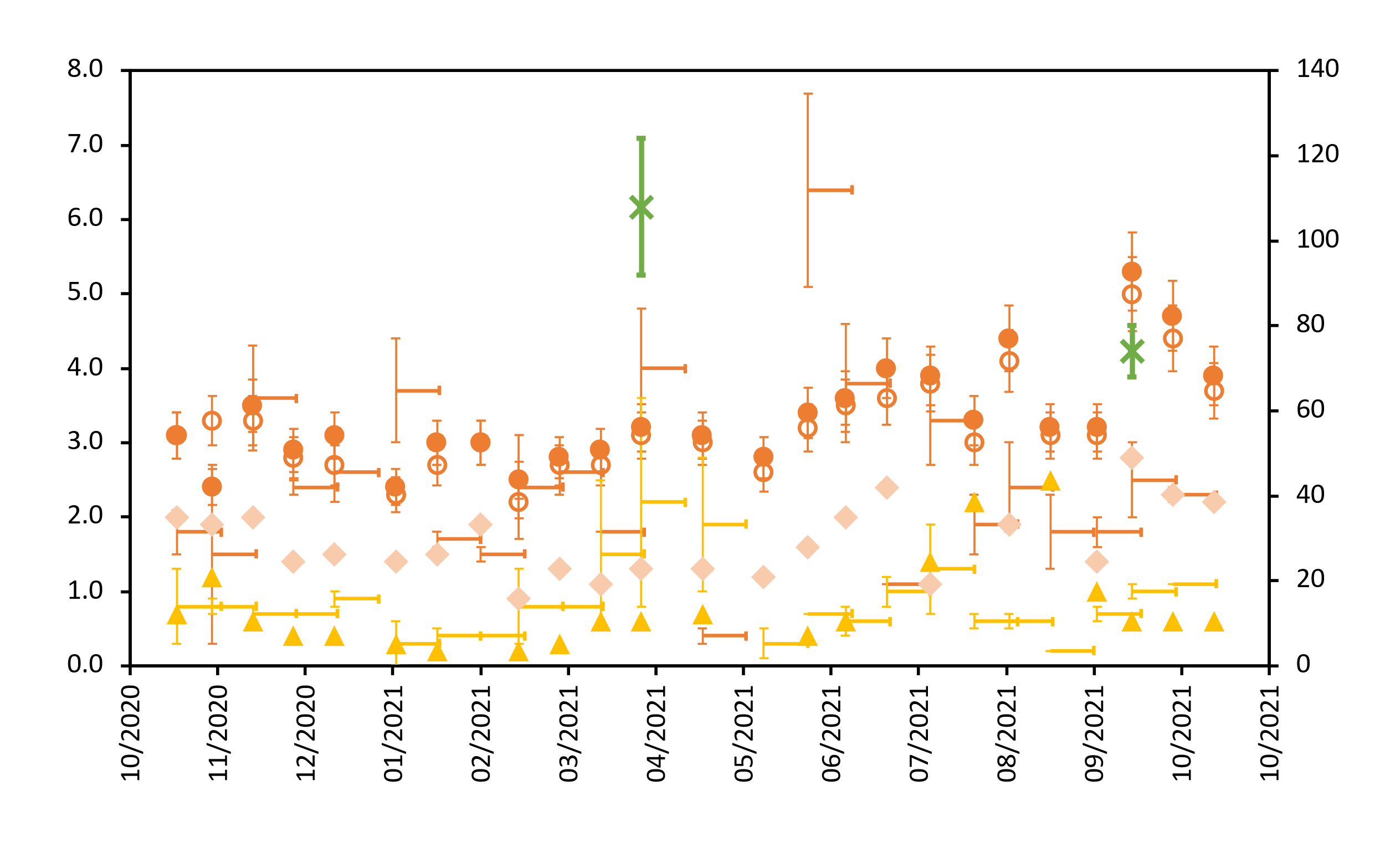

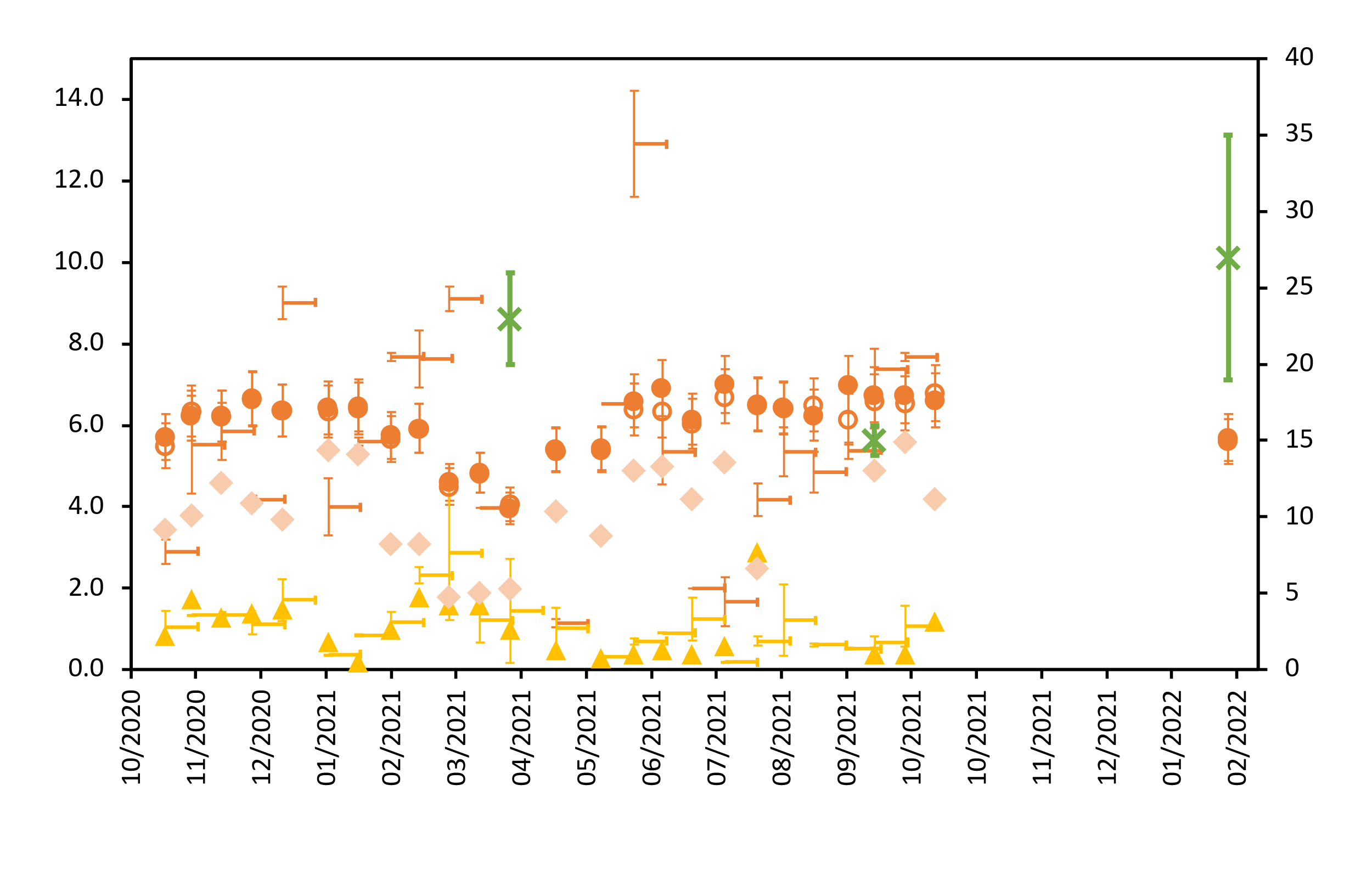

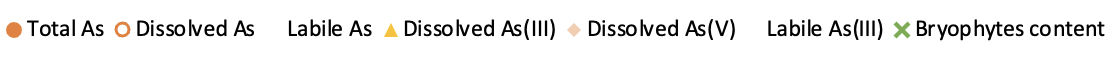

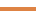

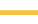


Site A1

Site A2

Site T1


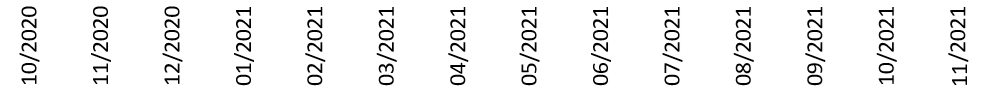


As concentrations in water (µg L^-1^)

As contents in bryophytes (mg kg^-1^_dry weight_)

Sampling period


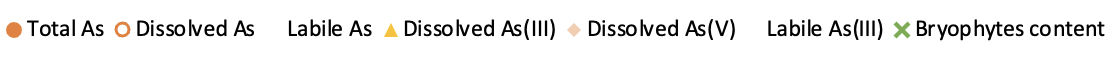

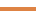

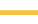


As concentrations in water (µg L^-1^)

As contents in bryophytes (mg kg^-1^_dry weight_)


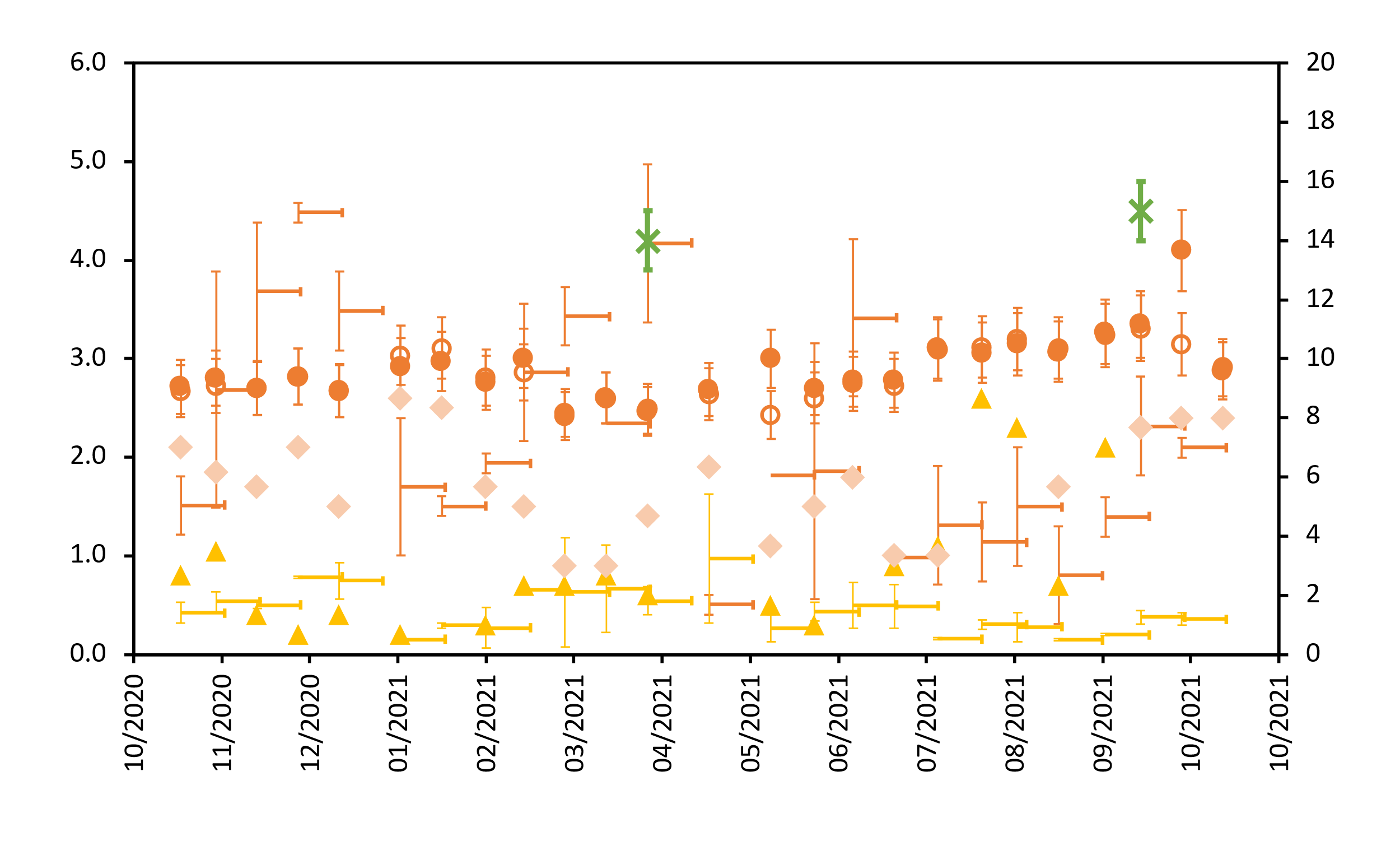

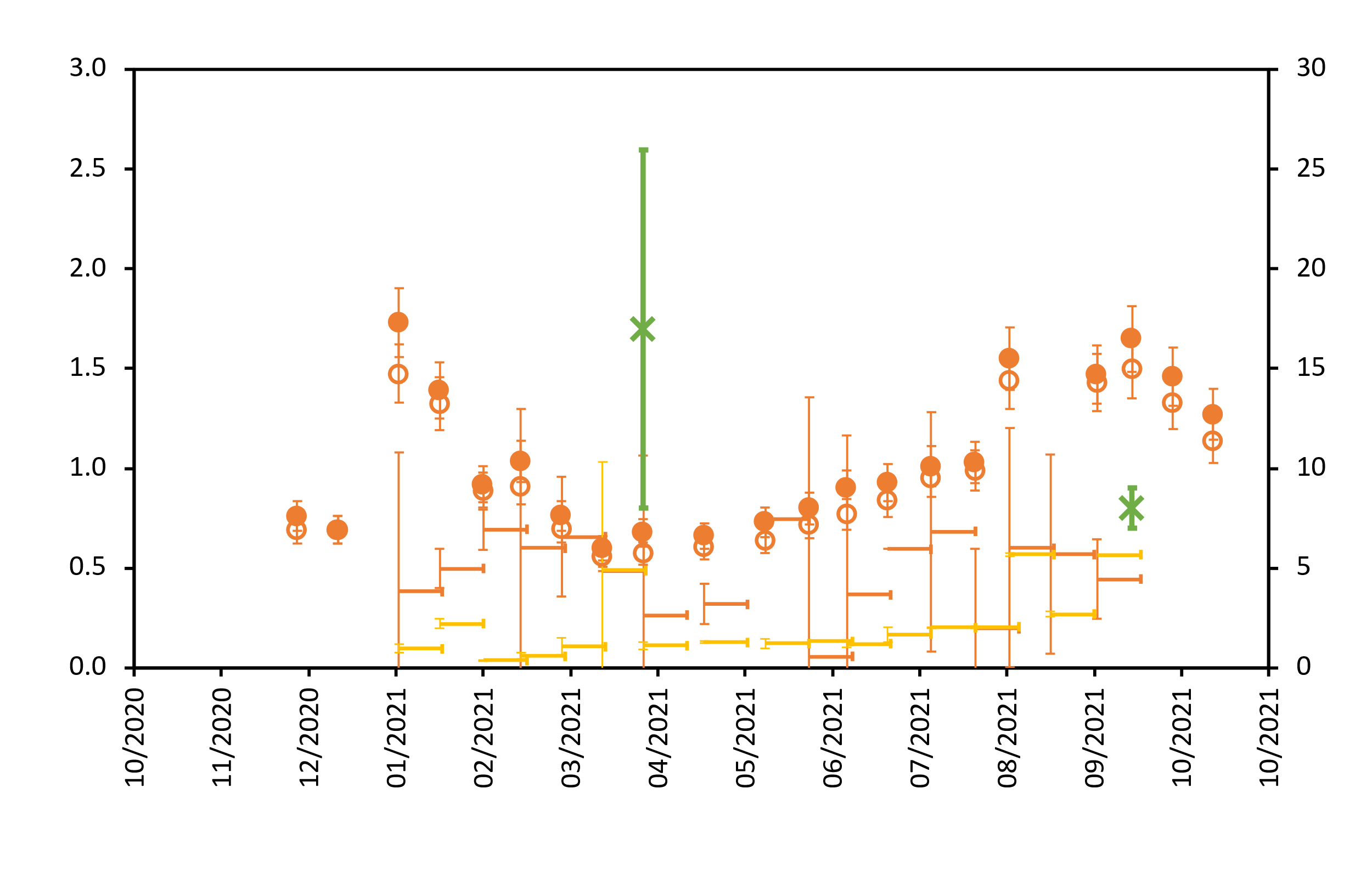

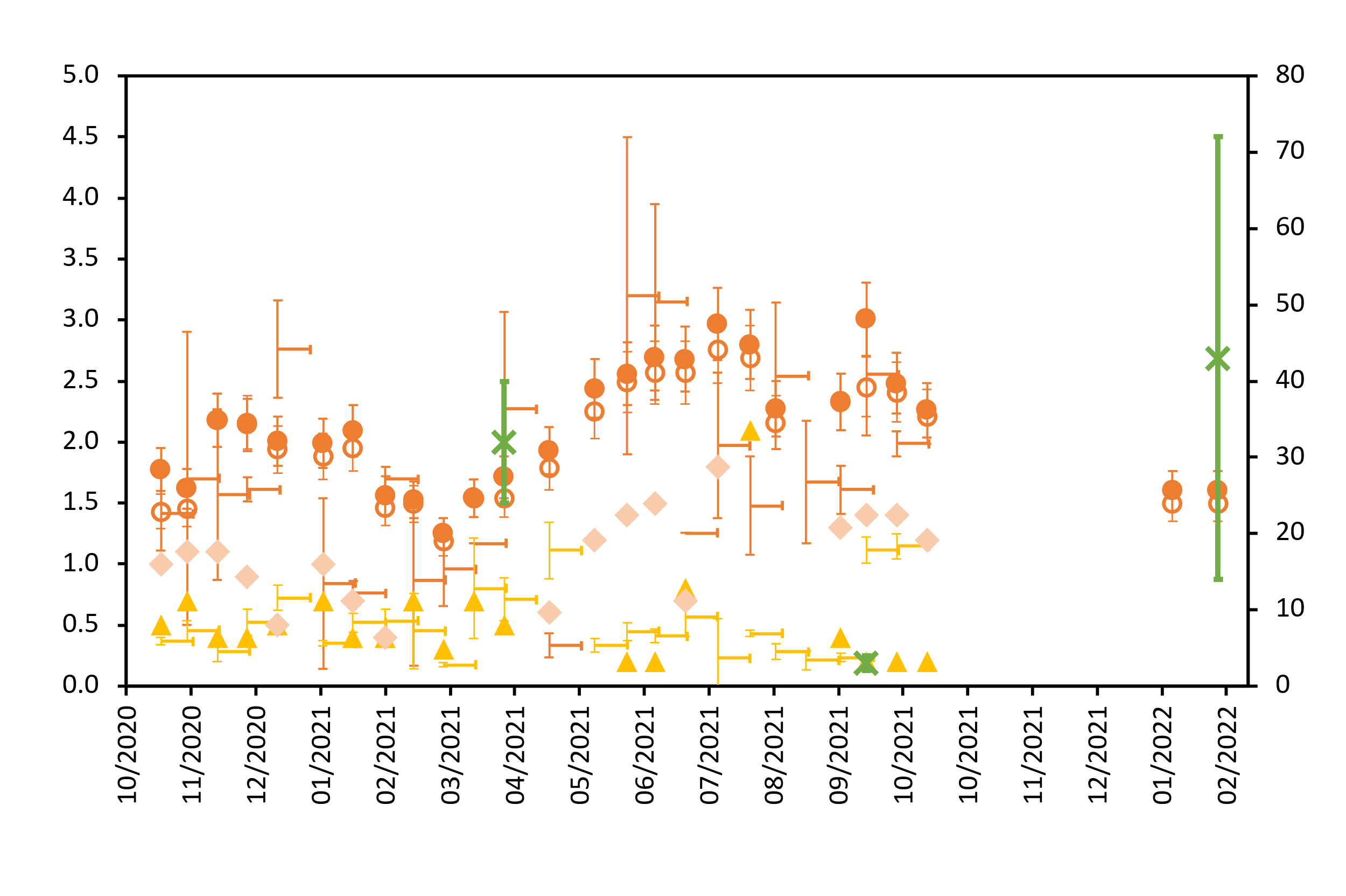

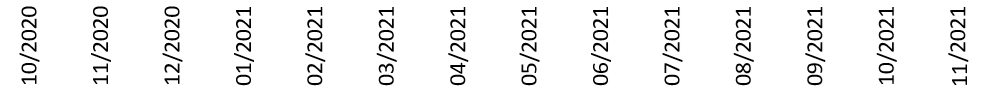

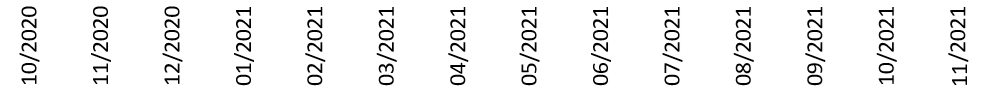


Sampling period

**Figure S1: Evolution of arsenic fractionation and speciation (grab and DGT sampling) over the one-year monitoring and arsenic contents in bryophytes at each sampling site** (mean value and standard deviation, n = 2)**.**

***Figure S2 : Unfavorable deployment conditions: low water level, high flow, and DGT placed on a sandy bed, leading to the intrusion of particles between the diffusive and binding gels (* photo of a binding gel during dismantling).***


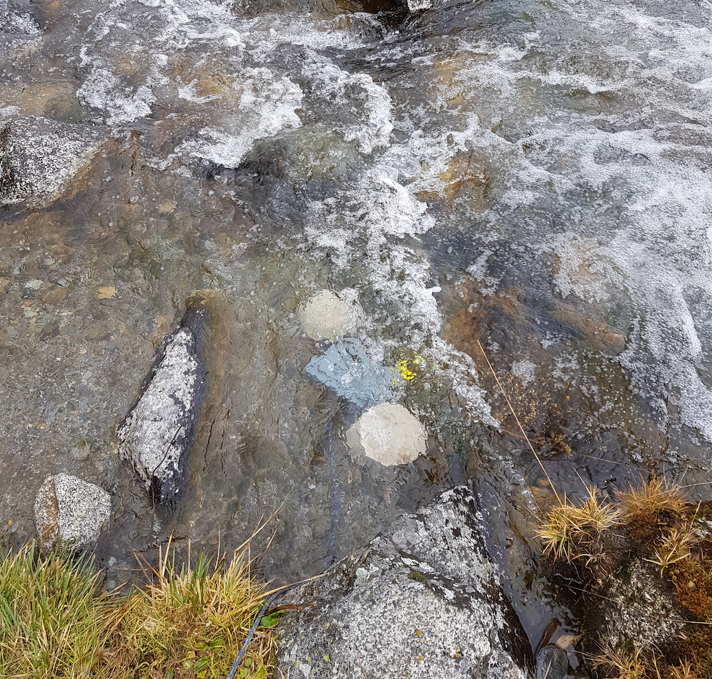

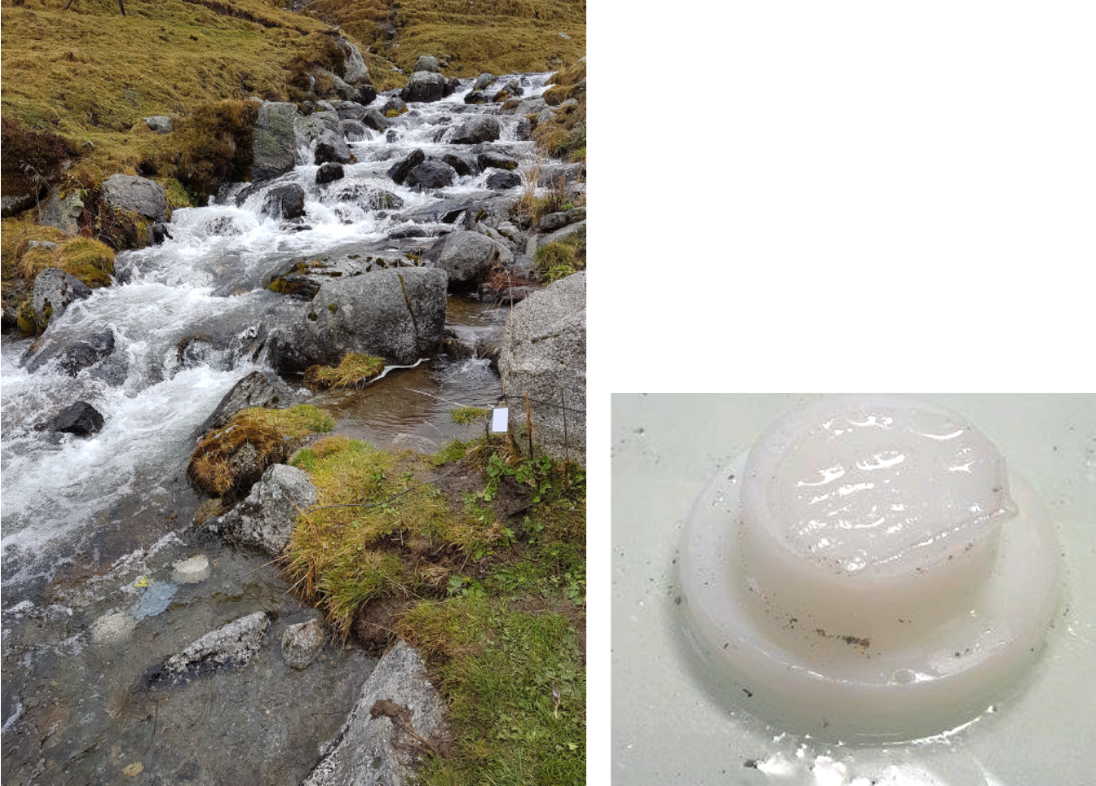


*

Particles entrapped in the device.

Particles in direct contact with the binding phase.
